# Impact of a Care Pathway for Older Patients Undergoing Emergency Abdominal Surgery: A Before‐and‐After Study

**DOI:** 10.1111/aas.70182

**Published:** 2026-01-09

**Authors:** Elin Kismul Aakre, Bjørn Steinar Olden Nedrebø, Atle Ulvik, Anette Hylen Ranhoff, Hans Flaatten, Karl Ove Hufthammer, Ib Jammer

**Affiliations:** ^1^ Department of Anaesthesia and Intensive Care Haukeland University Hospital Bergen Norway; ^2^ Department of Clinical Medicine University of Bergen Bergen Norway; ^3^ Department of Gastrointestinal Surgery Haukeland University Hospital Bergen Norway; ^4^ Department of Clinical Science University of Bergen Bergen Norway; ^5^ Centre for Clinical Research Haukeland University Hospital Bergen Norway; ^6^ Centre for Care Research West Western Norway University of Applied Sciences Bergen Norway

**Keywords:** acute perioperative care, emergency abdominal surgery, postoperative outcomes

## Abstract

**Background:**

Older patients undergoing emergency abdominal surgery face high risks of mortality and complications.

**Objective:**

Investigate whether a care pathway designed for older patients improves surgical outcomes.

**Methods:**

This single‐centre study investigated the effect of a care pathway for older patients undergoing emergency abdominal surgery, including preoperative frailty assessment, optimised perioperative care, and structured decision‐making for severely frail patients. Following implementation of the pathway, patients aged ≥ 75 years were prospectively enrolled in the ‘after’ cohort (1 January 2020–16 April 2021) and compared with a historical ‘before’ cohort of patients (1 January 2016–31 December 2017). The primary outcome was the Comprehensive Complication Index (CCI), a composite measure encompassing 30‐day mortality and postoperative complications. Secondary outcomes included 30‐day postoperative mortality, the number of palliative patients, mortality at 1 and 3 years and postoperative complications.

**Results:**

Among 154 patients in the ‘after’ cohort (median age 82 years [Q1–Q3: 78–86], 54% women, 53% frail), the primary outcome did not differ significantly from that of the 170 patients in the ‘before’ cohort (CCI mean [95% CI]: 44 [39–48] vs. 50 [46–55]; *p* = 0.15). Postoperative 30‐day mortality was significantly reduced (22% vs. 13%, *p* = 0.04). During the intervention, severely frail patients triaged to palliation (*n* = 12) were excluded from the study and received palliative care. High postoperative mortality was observed at 1 (31%) and 3 years (49%). Pulmonary (44% vs. 69%, *p* < 0.001) and gastrointestinal complications (39% vs. 52%; *p* = 0.02) were significantly reduced.

**Conclusion:**

In this ‘before‐and‐after’ study a care pathway designed for older patients undergoing emergency abdominal surgery had no significant impact on the composite outcome of postoperative mortality and complications. Postoperative 30‐day mortality, pulmonary and gastrointestinal complications were significantly reduced, while long‐term mortality remained high. Although the results should be interpreted with caution, they highlight the importance of careful preoperative evaluation.

In this study, outcomes of elderly patients undergoing emergency abdominal surgery were compared before and after implementation of a care pathway for elderly patients in a moderately sized single centre. Overall it is noted that implementation of this care pathway resulted in a decision to offer palliation instead of surgery to several patients. While the primary outcome of comprehensive complication index did not differ between the before‐and after groups, several secondary outcomes were improved in the after group. It should be noted however that multiple confounders could explain the difference, including selection bias of a more robust population offered surgery. The one‐and three year mortality of both groups was very high, stressing the importance of a careful and critical assessment of preoperative comorbidity and frailty, as well as the importance of ensuring that the treatment offered aligns with the overall goals of care for the individual patient.

## 1 Introduction

Emergency abdominal surgery (EAS) carries a high risk of mortality and complications among older patients [1]. More than half of patients undergoing EAS are aged ≥ 65 years [2], frequently presenting with frailty [3, 4, 5], multimorbidities [6] and deteriorating physiology [7]. For this group, quality care requires careful preoperative selection to avoid futile surgery and meticulous intraoperative management. This is challenging in the high‐pressure emergency setting, with limited information and insufficient time for optimisation. Additionally, surgeons and anaesthetists are trained to assess comorbidities, placing less emphasis on frailty and mobility, despite these variables being crucial for survival in the older population [8]. Guidelines recommend involvement by geriatricians [9]; however, this expertise is not consistently available. Although care pathways in EAS have demonstrated reduced mortality [10, 11, 12], the complex needs of older patients—integrating frailty evaluation, physiological assessment, and the exploration of patient preferences—have not yet been adequately addressed within these pathways.

A retrospective investigation from our hospital revealed a 26% 30‐day mortality rate among octogenarians who underwent EAS [13]. This finding demonstrated the need for a streamlined care pathway designed for older EAS patients, establishing a minimum standard of care while also integrating palliative options when appropriate. In the absence of a geriatric service within our institution, it was imperative for the pathway to be managed effectively by surgeons and anaesthetists.

Therefore, in close collaboration, surgeons, anaesthetists, intensivists, and geriatricians developed a care pathway aimed at improving the management of older patients requiring EAS. The pathway incorporated a structured preoperative assessment, a clinical decision‐making strategy for severely frail patients, and enhanced perioperative care.

The aim of the present study was to evaluate the effect of this care pathway, comparing our results with those of a historical ‘before’ cohort. Our primary outcome was the Comprehensive Complication Index (CCI), which is a composite score of 30‐day mortality and postoperative complications [14]. The secondary endpoints were (1) 30‐day postoperative mortality; (2) the number of palliative patients; (3) postoperative mortality at 1 and 3 years and (4) the incidence and nature of postoperative complications.

## 2 Methods

We conducted the study in line with the Helsinki Declaration, adhering to the STROBE guidelines for reporting observational studies [15]. The study was approved by the Regional Committee for Medical and Health Research Ethics (REK ID 2019‐7110) and registered at http://clinicaltrials.gov/ (clinical trials identifier NCT04293653).

### 2.1 Participants and Setting

This single‐centre study was conducted at a 1100‐bed Norwegian university hospital, serving a referral area of 500,000 inhabitants, with a gastrointestinal surgery unit handling emergency and elective abdominal surgery. No geriatric service was available in the institution. Eligible patients were aged ≥ 75 years, with an indication to undergo EAS, defined as any gastrointestinal pathology requiring laparotomy/laparoscopy, including cholecystectomy and repair of wound dehiscence, within the next 72 h. Exclusion criteria were vascular surgery, laparoscopic appendectomy, hernia surgery without laparotomy/scopy, palliative surgery for previously known inoperable malignancy, and former participation in the ‘after’ cohort.

The ‘before’ cohort consisted of historical patients from 1 January 2016 to 31 December 2017, identified by applying the inclusion and exclusion criteria to the electronic operation planner. These patients received ‘standard care’, as no standardised management protocol for EAS existed at the time.

In the prospective ‘after’ cohort, consecutive patients were enrolled from 1 January 2020 to 16 April 2021 and treated in accordance with the care pathway described below.

### 2.2 Frailty Scoring

Prior to the intervention period, all involved anaesthetists were trained to use the Clinical Frailty Scale (CFS) version 1, developed by the Canadian Study of Health and Aging [16]. This 9‐category scale classifies individuals from ‘very fit’ (score = 1) to ‘terminally ill’ (score = 9). Patients are further described as robust (scores 1–3), vulnerable (score 4), frail (score ≥ 5), and severely frail (score ≥ 7). The scale is validated for individuals aged ≥ 65 years and has been investigated in EAS patients [3, 17], and validated in intensive care [18].

The Eastern Cooperative Oncology Group performance status scale (ECOG) was included in the frailty assessment to emphasize ambulation as a key factor in postoperative recovery.

### 2.3 Care Pathway

Key components included preoperative discussion between the attending surgeon and anaesthetist, frailty assessment, and consultant‐led surgery and anaesthesia. In cases of suspected severe frailty, treatment options were comprehensively re‐evaluated using the decision‐making strategy outlined below.

Patients awaiting surgery were monitored on the ward using the National Early Warning Score (NEWS). If deterioration in vital signs was detected, they were transferred to the post‐anaesthesia care unit (PACU) for stabilisation.

Intraoperative and postoperative care followed standardised protocols, as outlined in Table 1. Postoperatively, patients were managed by anaesthetists in the PACU and discharged after a minimum of 12 h, at the discretion of the treating physician.

**TABLE 1:** Care pathway vs. standard care.

Care pathway (‘After’ cohort)	Standard care (‘Before’ cohort)
*Preoperative care* Involvement of specialists in surgery and anaesthesia Preoperative discussion between the surgeon and anaesthetist regarding: Surgical indication, comorbidities, and frailty status of the patient Monitoring of vital parameters in the ward using the National Early Warning Score (NEWS). If the NEWS score was ≥ 5, prompt surgery was warranted (if resources were available), otherwise, preoperative stabilization in the post anaesthesia care unit (PACU) was initiated A 24–7 available operating theatre for emergency cases Preoperative frailty scoring by the attending anaesthetist using the Clinical Frailty Scale (CFS) Palliative care as an alternative treatment in case of extreme frailty *Intraoperative anaesthetic management* Epidural analgesia, unless contraindicated Induction and maintenance agents determined at the discretion of attending anaesthetist Maintain mean arterial pressure (MAP) ≥ 65 mmHg Fluid management plan (Details, see Supporting Information) Transfusion threshold for haemoglobin concentration at 9 g/dL Monitoring of anaesthesia depth using the bispectral index (BIS), range of 45–65 *Postoperative care* Postoperative observation in the PACU ≥ 12 h Delirium screening Referral to a physiotherapist	*Preoperative care* Ad hoc specialist involvement Ad hoc communication between surgeon and anaesthetist No systematic monitoring of patient's vital parameters in the ward No designated operating theatre for emergency cases during normal working hours No frailty assessment performed *Intraoperative anaesthetic management* Epidural analgesia, unless contraindicated Perioperative care based on the anaesthetist's experience No guidelines regarding MAP, fluid management, anaesthesia depth control or transfusion thresholds *Postoperative care* Discharge from PACU at the discretion of the attending anaesthetist No delirium screening

### 2.4 Strategy for Clinical Decision‐Making in Severely Frail Patients

**FIGURE 1:**
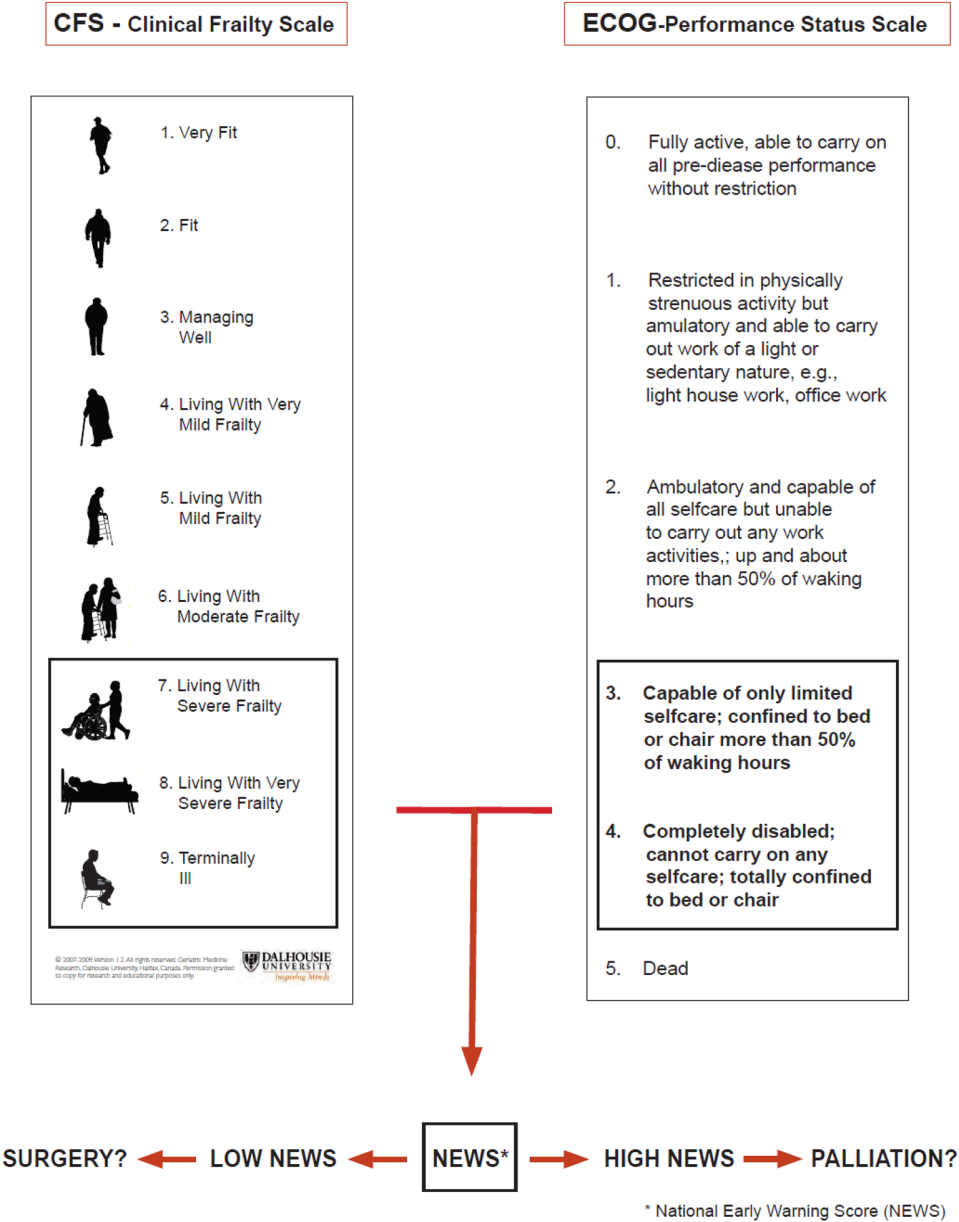
Strategy for clinical decision‐making in severely frail cases. Outline of the decision‐making strategy: to guide the choice of treatment plan, we evaluated frailty, mobility, and vital signs, together with the patient's expressed preferences. High frailty scores suggest limited capacity to recover from surgery, making palliative care a more appropriate option. We considered cut‐offs for frailty at CFS ≥ 7 and for immobility at ECOG ≥ 3. Acute physiological derangement was assessed using the NEWS score; however, no specific NEWS cut‐off was defined, as interpretation was left to clinical judgement. Hence, for patients with severe frailty (i.e., CFS ≥ 7) or significant immobility (i.e., ECOG ≥ 3), a second discussion on treatment approach was advised. Final treatment decisions were made on clinical grounds, guided by the strategy outlined in this figure, and through shared‐decision‐making with the patient and relatives.

The care pathway included a decision‐making strategy (Figure 1) to guide treatment for patients presenting with a CFS score of 7–9 and/or an ECOG score ≥ 3, indicative of severe frailty and a limited capacity to benefit from surgical intervention. Acute physiological derangement would further reduce the likelihood of a favourable surgical outcome; therefore, the NEWS score was also considered. Importantly, the choice of treatment should be made on clinical grounds, in collaboration with the patient and/or their relatives, and should be guided, not dictated, by the proposed strategy.

### 2.5 Consent

Informed consent was sought from all patients in the ‘after’ cohort, or from a relative in cognitively impaired patients, at the earliest possible opportunity following surgery. For the ‘before’ cohort, informed consent was waived, and the patients were informed about the study in accordance with the Norwegian General Data Protection Regulation.

### 2.6 Data Collection

In the ‘after’ cohort, all preoperative and intraoperative characteristics were collected prospectively, except for the Charlson Comorbidity Index [19] which was calculated retrospectively. The registration of comorbidities was based on the medical records. Data regarding cognitive impairment was based on information from relatives and the available medical records. In the ‘before’ cohort, all variables were collected retrospectively, and due to the risk of bias, retrospective frailty scoring was not performed. Postoperative complications during the hospital stay were collected retrospectively, with one investigator (EKAA) reviewing the medical records, classifying the complications according to standardised definitions [20], and grading severity according to the Clavien‐Dindo classification [21]. In the PACU, delirium screening was performed using the 4AT test [22]. As delirium screening tests were infrequently used at the wards, the documentation of delirium was based on the medical records, a method previously validated [23]. Major adverse cardiac events (MACE) were defined as myocardial infarction, non‐fatal cardiac arrest or coronary revascularisation [24]. The impact of each complication, including mortality, was calculated using the Comprehensive Complication Index (CCI) [14], ranging from 0 to 100. A score of 0 indicates an uneventful recovery, and 100 indicates death. Length of stay (LOS) was calculated as the number of days between admission and discharge.

### 2.7 Statistical Analysis

Sample size calculation was based on a previous investigation conducted at our hospital [13], from which we calculated the composite score of complications and 30‐day mortality in patients aged ≥ 80 years using the CCI. The mean CCI value was 42, and we considered a 25% reduction to be clinically significant. Based on the distribution of CCI scores in the historical data and clinical judgment, we postulated a distribution of CCI score consistent with an approximately 25% reduction in the mean score. To detect this reduction with 90% power at a 5% significance level using the Wilcoxon–Mann–Whitney, computer simulations showed that a total of 280 patients were sufficient, with 140 patients allocated to each group. Details are shown in the Supporting Information.

Categorical data are presented as counts and percentages and were compared using the chi‐squared test. Continuous data are presented as means, standard deviations (SD), and/or confidence intervals (CIs), or as medians with 25th and 75th percentiles and were compared using either Welch's two‐sided *t*‐test or the Mann–Whitney test.

We present the mortality data using Kaplan–Meier plots. To compare the mortality in the ‘after’ cohort and historical ‘before’ cohort, we used the log‐rank test with administrative censoring at either 30 days, 1 year (365 days) or 3 years (1095 days). All patients were followed for at least 3 years, with no other censoring. To compare the mortality of the ‘after’ cohort with that of the general population, we calculated the expected survival for a similar sample based on age‐ and sex‐specific life tables for the general Norwegian population. The life tables were obtained from the Human Mortality Database [25] and are originally based on mortality data from Statistics Norway. To calculate the expected survival, we used the Hakulinen method, with expected follow‐up set to 3 years. We also report the one‐ and three‐year standardised mortality ratio (SMR).

*p* values ≤ 0.05 are characterised as statistically significant, and all CIs reported are 95% CIs. The statistical analyses were performed using either SPSS version 24 software (Chicago, IL, USA) or R version 4.4.1 [26]. Due to a low number of missing data (Table S4), we used complete‐case analysis.

## 3 Results

Of the 476 screened patients, 324 were included: 170 in the historical ‘before’ cohort and 154 in the ‘after’ cohort (Figure S1). The groups were comparable in terms of age, comorbidity burden, and preoperative habitation status, except for patients residing in a nursing home prior to surgery (3% in the ‘after’ cohort compared to 9% in the ‘before’ cohort, SMD 0.25; Table 2). Of the total population (*n* = 324), more than half (61%) were aged 80 years or older. Serious comorbidities were prevalent, with 25% of patients having chronic obstructive pulmonary disease, 47% reduced kidney function, and a median of five daily prescriptions. A small proportion of patients were admitted from a nursing home or assisted living facility (15%). Among those living at home (*n* = 276), 18% received daily assistance from healthcare services. In the ‘after’ cohort, 53% of patients were classified as either vulnerable or frail (CFS ≥ 4).

**TABLE 2:** Patient characteristics.

Characteristics	‘After’ cohort *n* = 154	‘Before’ cohort *n* = 170	SMD	*p*
Age (years), median (Q1–Q3), range	82 (78–86), 75–97	81 (78–85), 75–93	0.11	0.54^a^
Patients aged ≥ 90	20 (13)	12 (7)	0.20	0.07^b^
Sex ratio (M:F)	71:83	74:96	0.05	0.64^b^
American Society of Anaesthesiologists' Physical Status Classification (ASA Classification)				0.60^b^
ASA class 1	1 (0)	0 (0)	0.11	—
ASA class 2	27 (18)	24 (14)	0.09	—
ASA class 3	92 (60)	104 (61)	0.03	—
ASA class 4	34 (22)	41 (24)	0.05	—
ASA class 5	0 (0)	1 (1)	0.11	—
ASA class (continuous)	3.0	3.1	0.12	—
Preoperative NEWS score, median (Q1–Q3)	3 (1–5)	—	—	0.32^a^
Comorbidities				
Chronic obstructive pulmonary disease (COPD)/asthma	40 (26)	40 (24)	0.06	0.61^b^
Cognitive dysfunction or dementia	22 (14)	15 (9)	0.17	0.12^b^
Glomerular filtration rate < 60 mL/min	76 (49)	75 (44)	0.11	0.35^b^
Charlson Comorbidity Index, median (Q1–Q3)	5 (4–6)	5 (4–7)	0.18	0.18^a^
Daily medication, median (Q1–Q3)	5 (4–7)	5 (2–8)	0.06	0.19^a^
Pre‐operative habitat				0.16^b^
Own home without daily help from healthcare personnel	111 (72)	116 (68)	0.08	—
Own home with daily help from healthcare personnel	25 (16)	24 (14)	0.06	—
Assisted living facility	13 (8)	14 (8)	0.01	—
Nursing home	5 (3)	16 (9)	0.25	—
Clinical frailty scale (CFS) score				
CFS score 1	11 (7)	—	—	—
CFS score 2	23 (15)	—	—	—
CFS score 3	38 (25)	—	—	—
CFS score 4	41 (27)	—	—	—
CFS score 5	16 (10)	—	—	—
CFS score 6	21 (14)	—	—	—
CFS score 7	4 (3)	—	—	—
Eastern Cooperative Oncology Group performance (ECOG) status				
ECOG Status 0	33 (10)	—	—	—
ECOG Status 1	37 (11)	—	—	—
ECOG Status 2	39 (12)	—	—	—
ECOG Status 3	44 (14)	—	—	—
ECOG Status 4	1 (0)	—	—	—

*Note:* Values are *n* (%) unless indicated otherwise.

Abbreviation: SMD, standardised mean difference.

^a^ Mann–Whitney test.

^b^ Chi‐square test.

Surgical indications and intraoperative findings were comparable between the groups (Table S1), with procedural details provided in Table S2. In the ‘after’ cohort, a higher proportion of patients underwent laparoscopic procedures (21% vs. 11%; *p* = 0.01) and fewer surgeries occurred outside regular hours (58% vs. 71%; *p* = 0.02). The ‘after’ cohort had more often consultant involvement, more frequent anaesthesia depth monitoring, and longer PACU stays (Table S4). Perioperative care parameters—such as fluid management, vasopressor use, and physiotherapy referral—were largely similar (Tables S3 and S4). Adherence rates to the elements of the care pathway were high, predominantly exceeding 80% (Table S4).

The primary outcome of composite 30‐day mortality and complications for the ‘after’ cohort was mean 44 (SD 28; 95% CI 39–48). This was not statistically significantly different from the ‘before’ cohort, mean 50 (SD 31; 95% CI 46–55), *p* = 0.15 (Table 3).

**TABLE 3:** Postoperative complications, length of stay and 30‐day readmissions.

Complication	‘After’ cohort *n* = 154		‘Before’ cohort *n* = 170		Difference (‘Before’–‘After’)		
	Est. (*n*)	Prop.	Est. (*n*)	Prop.	Est.	95% CI^a^	*p*
Comprehensive complication index							0.15^b^
Mean (SD; 95% CI)	44	(28; 39–48)	50	(31; 46–55)	−6	−13 to 0	—
Median (Q1–Q3)	40	(23–58)	41	(30–71)	1	—	—
30‐day mortality	20	13%	37	22%	9%	1%–17%	0.04^c^
30‐day mortality in patients with indication for surgery, *n*/number at risk	32/166	19%	—	—	—	—	—
Postoperative pulmonary complications (POPC)	68	44%	117	69%	25%	14%–35%	< 0.001^d^
Acute kidney injury							0.02^d^
No kidney injury	84	55%	99	58%	4%	−7% to 14%	0.50^d^
Stage 1	19	12%	36	21%	9%	1%–17%	0.03^d^
Stage 2	38	25%	22	13%	−12%	−20% to −3%	0.007^d^
Stage 3	13	8%	13	8%	−1%	−7% to 5%	0.79^d^
Delirium	64	42%	67	39%	−2%	−13% to 9%	0.69^d^
Gastrointestinal complications (ileus, obstipation, and diarrhoea)	60	39%	88	52%	13%	2%–24%	0.02^d^
Wound infection (superficial/fascial dehiscence/abscess)	23	15%	17	10%	−5%	−12% to 2%	0.18^d^
Anastomotic breakdown	5	3%	1	1%	−3%	−6% to 0%	0.08^d^
Major adverse cardiac event	5	3%	8	5%	1%	−3% to 6%	0.50^d^
Multiorgan failure	12	8%	21	12%	5%	−2% to 11%	0.18^d^
Pneumonia	10	6%	7	4%	−2%	−7% to 3%	0.34^d^
Infection, source unknown	4	3%	4	2%	0%	−4% to 3%	0.89^d^
Urinary tract infection	2	1%	1	1%	−1%	−3% to 1%	0.50^d^
Stroke	2	1%	1	1%	−1%	−3% to 1%	0.50^d^
Pulmonary embolism	7	5%	5	3%	−2%	−6% to 3%	0.45^d^
Gastrointestinal bleeding	0	0%	2	1%	1%	0%–3%	0.18^d^
Red blood cell transfusion	61	40%	56	33%	−7%	−17% to 4%	0.21^d^
Total parenteral nutrition	63	41%	55	32%	−9%	−19% to 2%	0.11^d^
Reoperation	20	13%	23	14%	1%	−7% to 8%	0.89^d^
Re‐admission to ICU	0	0%	4	2%	2%	0%–5%	0.06^d^
Length of stay (days)^e^, median (Q1–Q3) (*n* = 263)	10	(7–15)	11	(8–21)	1	—	0.03^b^
Discharge destination nursing home, *n*/number at risk^f^	57/135	42%	68/133	51%	9%	−3% to 20%	0.16^d^
30‐day readmission, *n*/number at risk^f^ (%)	40/135	30%	26/133	20%	−10%	−20% to 0%	0.06^d^
Long‐term outcome							
1‐year mortality	48	31%	68	40%	9%	−2% to 19%	0.08^c^
3‐year mortality	76	49%	101	59%	10%	−1% to 21%	0.04^c^

^a^ Wald confidence interval for proportions; based on *t*‐test for continuous data.

^b^ Mann–Whitney test.

^c^ Log‐rank test.

^d^ Chi‐squared test.

^e^ Calculated as days between admission and discharge.

^f^ Number at risk are those discharged from hospital alive.

Postoperative 30‐day mortality was 13% in the ‘after’ cohort, compared to 22% in the ‘before’ cohort (*p* = 0.04; Table 3). During the intervention period, 12 patients were deemed too frail for surgery and received palliative care (Figure S1). These patients were excluded from the ‘after’ cohort and the statistical analyses. To illustrate the effect of triage, we report mortality in the ‘after’ cohort including the 12 patients triaged to palliative care (Table 3), assuming they died within 30 days. The patients in the ‘before’ cohort were identified through the surgical booking system and had all undergone surgery; therefore, the number of palliative patients during this period is unknown.

**FIGURE 2:**
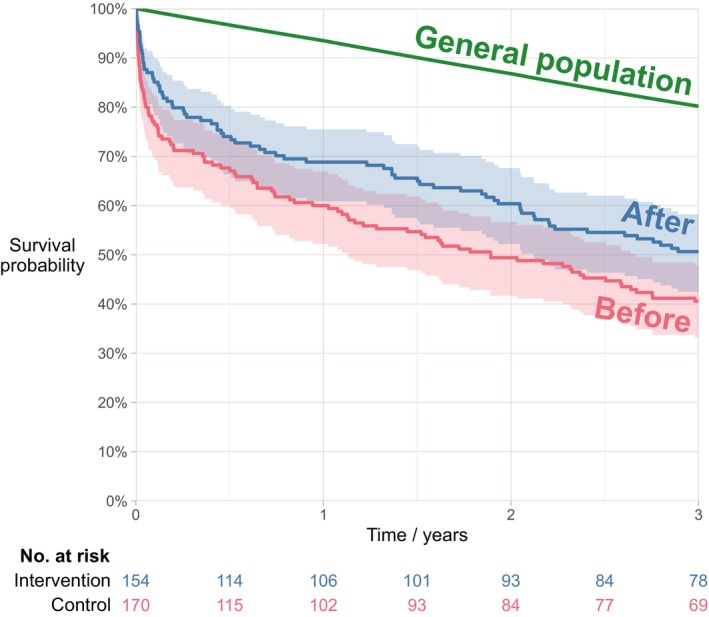
Kaplan–Meier survival curve for up to 3 years following emergency abdominal surgery. Three‐year survival following emergency abdominal surgery (patients aged 75 years or older). The coloured bands show 95% point wise confidence intervals. The operated patients are compared to the general Norwegian population, matched by age and sex. The standardised mortality ratio was 4.8 (95% CI: 3.5–6.3) at 1 year and 2.5 (95% CI: 2.0–3.1) at 3 years.

One‐year postoperative mortality was 31% in the ‘after’ cohort, compared to 40% in the ‘before’ cohort (*p* = 0.08). Three‐year mortality was 49% and 59%, respectively (*p* = 0.04; see Table 3 or Figure 2). Compared to the corresponding age‐ and sex‐standardised general Norwegian population, patients exhibited nearly fivefold higher mortality (Figure 2). One year after surgery, 48 deaths had occurred in the ‘after’ cohort, compared to an ‘expected’ 10.1 (SMR 4.8; CI 3.5–6.3, *p* < 0.001). Three years after surgery, 76 deaths had occurred in the ‘after’ cohort, compared to an ‘expected’ 30.6 (SMR 2.5; CI 2.0–3.1, *p* < 0.001).

The complications occurring most frequently in both groups were postoperative pulmonary complications (POPC), acute kidney injury, delirium, and complications related to gastrointestinal tract passage (Table 3). In the ‘after’ cohort, there were significantly fewer pulmonary (44% vs. 69%, *p* < 0.001) and gastrointestinal complications (39% vs. 52%, *p* = 0.02), and median LOS was significantly reduced (10 days vs. 11 days; *p* = 0.03). The 30‐day readmission rate was highest in the ‘after’ cohort (30% vs. 20%; *p* = 0.06). Nearly half of the patients in both groups were discharged to a nursing home (42% vs. 51%; *p* = 0.16).

## 4 Discussion

Given the high mortality following EAS in older patients [1], surprisingly few studies have investigated the impact of targeted interventions aimed at this population. To our knowledge, the present study is the first to explore the effects of a structured care pathway in older EAS patients. We observed no statistically significant impact of the care pathway on the composite of 30‐day mortality and complications, and the long‐term outcomes were discouraging, despite the increased focus and enhanced management with the care pathway. Nevertheless, our findings suggest that the care pathway may reduce postoperative risks, although the 31% one‐year mortality underscores the need for careful preoperative patient selection.

To our knowledge, there is only one previous study published of a care pathway for older EAS patients, investigating a postoperative care pathway with geriatric services. That study demonstrated a 19% absolute reduction in complications and mortality [27]. This population was younger (mean age 76 years) and likely more robust, as nursing home residents and patients with CFS score ≥ 7 were excluded. Additionally, approximately one‐third of the participants underwent only minor procedures.

A meta‐analysis investigating care pathways in the adult EAS population indicated a reduction in postoperative complications but found no significant impact on mortality [28]. Compared to our study, the patients were much younger, mean 35–65.8 or median 33–75 years.

Thirty‐day postoperative mortality was significantly lower in the ‘after’ cohort (13%) compared to the ‘before’ cohort (22%; *p* = 0.04). The improved survival among those undergoing surgery may reflect the intentional selection of robust patients, as the decision‐making strategy promoted modification of treatment plans from surgery to palliation when severe frailty was detected. Improved survival may also be due to improved management through the care pathway, including more consultant involvement, more laparoscopic procedures, and increased daytime surgeries. Unfortunately, the number of palliative patients during the control phase is unknown.

Our observed mortality rates at one (31%) and 3 years (49%) align with those of a Swedish study reporting a 1‐year mortality of 29.6% in patients aged ≥ 76 years [29]. Our findings are also comparable with a systematic review [30] involving 1187 patients aged (median) 79–85 years, reporting 1‐year mortality between 30% and 47%. The long‐term mortality in our study was surprisingly high compared to the general population, with a standardised mortality ratio of 4.8 at 1 year and 2.5 at 3 years. The high long‐term mortality underscores the need for thorough preoperative selection of robust individuals for surgery.

Evidence concerning postoperative complications after EAS is limited [27, 31]. In our study, the predominant morbidities after surgery included POPCs, gastrointestinal complications, kidney failure, and delirium, which align with findings from other reports [31]. The care pathway significantly reduced the proportion of POPCs, gastrointestinal complications, and the median LOS. These improvements may be explained by improved perioperative care, a higher rate of laparoscopic procedures, as well as preoperative triage favouring patients better able to tolerate postoperative functional decline.

Balancing benefit and futility in high‐risk procedures for older patients is challenging, and care pathways should incorporate approaches to recognize treatment futility. Studies indicate that invasive procedures are offered to extremely frail patients, leading to excess mortality [7, 32]. On the other hand, in patients deemed too frail for EAS, palliative management may yield outcomes more favourable than anticipated. In a study involving 750 patients indicated for emergency laparotomy who were palliated due to presumed futility, one‐fifth were still alive at 90 days [33]. This unexpected finding highlights the complexity of decision‐making in frail patients requiring EAS. Consequently, care pathways should acknowledge that for the frailest, death is not always perceived as the worst outcome [34, 35].

### 4.1 Strengths of the Study

This study is probably the first to explore the impact of a structured care pathway designed for older EAS patients. Our inclusion criteria were broad, allowing us to include nearly the entire population of older EAS patients at our hospital, thus increasing the validity of our findings. Additionally, we performed a comprehensive frailty assessment. Furthermore, the simplicity of the care pathway, with no special equipment requirements, enhances its potential for broad implementation. The mortality data were retrieved from the Norwegian National Population Registry, facilitating easy and reliable long‐term follow‐up.

### 4.2 Limitations of the Study

There is a high risk of selection bias between the two cohorts. Patients in the ‘before’ cohort were identified via the surgical booking system and had all undergone surgery. The number of patients triaged to palliation in this era (2016–2017) cannot be determined in retrospect. By contrast, the ‘after’ cohort involved deliberate selection of the most robust patients. Consequently, the effect of the care pathway is likely overestimated, and our findings should be interpreted with caution. Moreover, the time interval between the cohorts may have influenced the outcomes observed in the ‘after’ cohort. During this interval, Enhanced Recovery After Surgery (ERAS) protocols were implemented for elective colorectal procedures, and NEWS monitoring was introduced on the wards. In addition, the surgeons' competence in laparoscopic techniques improved over time, leading to a higher proportion of laparoscopic procedures in the ‘after’ cohort.

Furthermore, during the intervention phase the staff were aware of the ongoing study, potentially introducing performance bias.

The review of postoperative complications by a single investigator may have affected data consistency, despite adherence to standardised definitions. The Norwegian healthcare context may also limit the generalisability of these findings. The ongoing COVID‐19 pandemic during the intervention period may have influenced our results, as the volume of elective surgical cases declined, while surgical ward nurses were not reassigned to other departments. Consequently, the patient‐to‐nurse staffing ratio was better than usual.

Further research should focus on patient‐reported outcomes following EAS and explore interventions aimed at mitigating postoperative complications. Patients deemed unfit for surgery warrant further investigation.

## 5 Conclusion

This ‘before and after’ study evaluated a care pathway for older EAS patients, incorporating frailty assessment, optimised perioperative care and pre‐operative triage to palliation. Although the findings should be interpreted with caution, we observed no significant reduction in the composite outcome of 30‐day mortality and complications. Postoperative 30‐day mortality, pulmonary and gastrointestinal complications were significantly reduced. Mortality remained high at 1 year (31%) and 3 years (49%), highlighting the importance of careful patient selection before surgery.

## Author Contributions

**Elin Kismul Aakre:** conceived of the presented idea, recruited the patients, contributed to data analysis and interpretation, wrote the first draft of the manuscript, and approved the final version. **Bjørn Steinar Olden Nedrebø:** contributed to conceptualizing the study, contributed to the interpretation of the data, was involved in drafting and critically revising the manuscript, and approved the final version. **Atle Ulvik:** contributed to conceptualizing the study, contributed to the interpretation of the data, was involved in drafting and critically revising the manuscript, and approved the final version. **Anette Hylen Ranhoff:** contributed to conceptualizing the study, was involved in drafting and critically revising the manuscript, and approved the final version. **Hans Flaatten:** contributed to conceptualizing the study, was involved in drafting and critically revising the manuscript, and approved the final version. **Karl Ove Hufthammer:** oversaw and completed the statistical analysis and was involved in drafting and critically revising the manuscript and approved the final version. **Ib Jammer:** conceived of the presented idea, was involved in drafting and critically revising the manuscript, and approved the final version.

## Funding

This work was supported by Helse Vest (F‐11005).

## Disclosure

The results were presented at the Annual Conference of Norwegian Anaesthetists, ‘Høstmøtet 2023’, abstract number 14 (available in Norwegian only), and at the annual conference of European Anaesthetists, ‘Euroanesthesia 2024’, abstract reference 22AP03‐05, available from https://esaic.org/wp‐content/uploads/2024/06/esaic2024_abstracts.pdf.

## Ethics Statement

The study was approved by the Norwegian Regional Ethics Committee (REK ID 2019‐7110).

## Conflicts of Interest

Elin Kismul Aakre, Bjørn Steinar Olden Nedrebø, Atle Ulvik, Anette Hylen Ranhoff, Hans Flaatten, and Karl Ove Hufthammer declare no conflicts of interest. Ib Jammer reports research grants from the European Society of Anaesthesiology and Intensive Care (ESAIC) and Roche and speaker honoraria from Orion and is chair of the Perioperative Medicine and Management (PoMM) program of the Scandinavian Society of Anaesthesia and Intensive Care (SSAI).

## Supporting information


**Figure S1:** Patient flow diagram.
**Table S1:** Perioperative logistic, indications for surgery and intraoperative variables.
**Table S2:** Surgical procedures.
**Table S3:** Intraoperative fluid therapy.
**Table S4:** Adherence to bundle care elements.

## Data Availability

The data that support the findings of this study are available from the corresponding author upon reasonable request.
