# Supplementary material for: Impact of a Care Pathway for Older Patients Undergoing Emergency Abdominal Surgery: A Before‐and‐After Study
Source: Acta Anaesthesiol Scand. 2026 Jan 9;70(2):e70182. doi: 10.1111/aas.70182 (PMC12789884; doi:10.1111/aas.70182)
Supplement: Supplementary file 1 — Figure S1: Patient flow diagram. Table S1: Perioperative logistic, indications for surgery and intraoperative variables. Table S2: Surgical procedures. Table S3: Intraoperative fluid therapy. Table S4: Adherence to bundle care elements. [file AAS-70-0-s001.pdf]

Supplementary material

Contents:

Figure S1: Patient flow diagram .... Page 2

Table S1: Periop. logistic, indications for surgery, intraop. variables .... Page 3

Table S2: Surgical procedures .... Page 4

Intraoperative fluid management plan (‘After’cohort) .... Page 5

Table S3: Intraoperative fluid therapy .... Page 5

Table S4: Adherence to bundle care elements .... Page 6

Sample size considerations .... Page 7

Figure S1: Patient flow diagram

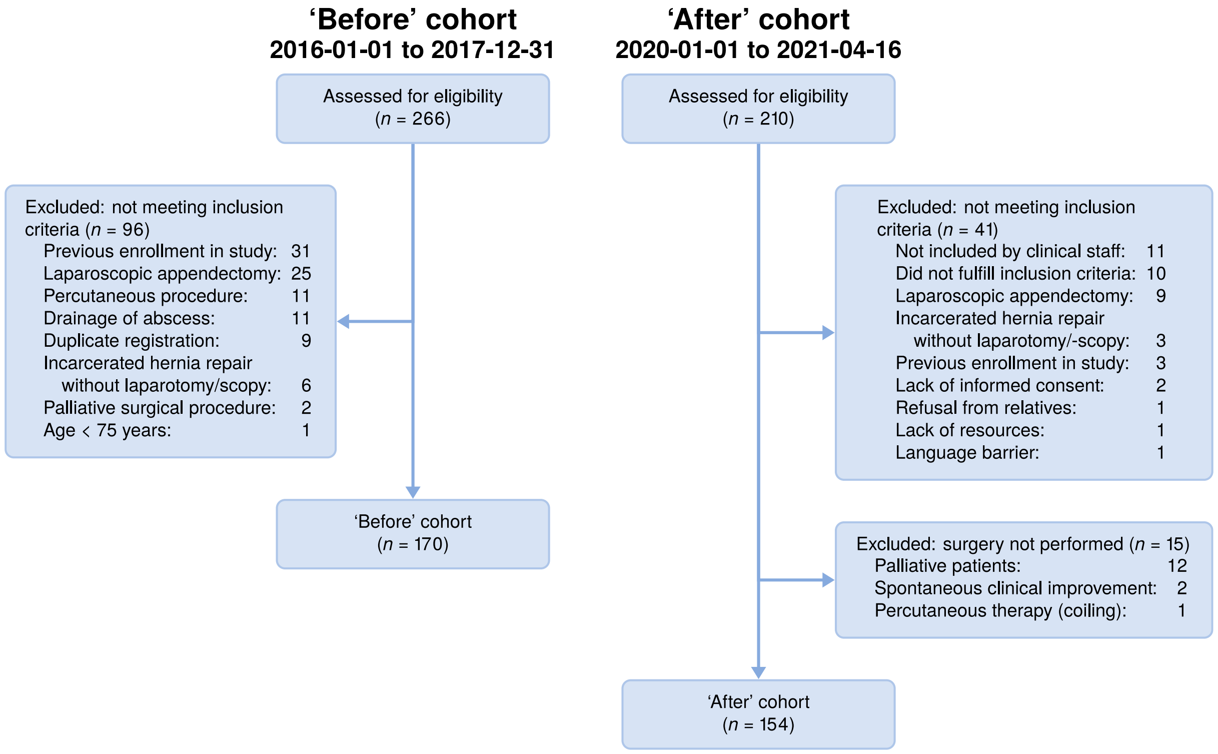

Table S1: Perioperative logistic, indications for surgery and intraoperative variables

Variable	'After' cohort n=154	'Before' cohort n=170	*P*-value
**Perioperative logistic**			
Pre-op Ct, n (%)	145 (94)	154 (91)	0.23[b]
Pre-op stabilization, n (%)	20 (13)	19 (11)	0.62[b]
Time from admittance to CT scan, h	7 (2-21)	12 (4-44)	0.04[a]
Time from CT scan to start of surgery, h	10 (4-26)	10 (4-23)	0.48[a]
Surgery performed after normal working hours (after 16:00, or on holidays and weekends)	89 (58)	120 (71)	0.02[b]
**Indications for surgery**			0.17[b]
Obstruction, n (%)	92 (60)	83 (49)	
Perforation, n (%)	32 (21)	36 (21)	
Sepsis, n (%)	20 (13)	25 (15)	
Ischemia, n (%)	5 (3)	14 (8)	
Bleeding, n (%)	3 (2)	6(4)	
Other (wound dehiscence, planned relook), n (%)	2 (1)	6 (4)	
**Surgical procedures** *(For further details, see Table S2)*			
Laparoscopy (completed), n (%)	32 (21)	18 (11)	0.01[b]
Laparotomy, n (%)	122 (79)	152 (89)	
Laparoscopy intended and converted, n (%)	20 (13)	11 (7)	0.05[b]
Duration of surgery, mean (SD), min	129 (66)	120 (63)	0.21[c]
**Intraoperative findings**			
Perop cancer, n (%)	37 (24)	47 (28)	0.46[b]
Perop blood loss, ml	100 (50-150)	100 (50-163)	0.25[a]
Soiling (pus, blood), n (%)	54 (35)	62 (37)	0.79[b]

Values are median and Q1-Q3 unless otherwise indicated. Numbers are reported as n (%)

[a] Mann–Whitney test

[b] Chi-squared test

[c] *t*-test

Table S2: Surgical procedures

Surgical procedure	'After' cohort (n=154)	'Before' cohort (n=170)
**Bowel resection**		
Colon	32	40
Small intestine	37	28
Colon and small intestine	4	8
Rectum	4	7
Duodenum	1	0
Pyloroplasty	1	1
Stomach	4	2
Adhesiolysis	30	46
Appendectomy (open incision)	4	4
**Stoma**		
Stoma formation	22	39
Stoma revision	3	1
Lavage (laparotomy or laparoscopy)	12	16
**Closure of perforation**		
Esophagus	0	1
Stomach (gastroraphy)	3	11
Duodenum	3	5
Enteroraphy	0	12
**Hernia repair**		
Groin/femoral/umbilical	7	5
Hernia through diaphragma	0	3
**Exploratory procedure**		
Laparotomy	3	4
Laparoscopy	1	2
Removal of foreign object (through enterotomy)	4	0
**Other procedures**		
Entero-enteroanastomosis or gastro-enteroanastomosis	4	10
Cholecystectomy (open or laparoscopic)	6	3
Repair of abdominal wound dehiscence	3	6
Other	1	2
**Number of procedures***	189	256
Re-operation after elective surgery	16	23

*Number of procedures exceeds number of individuals as several procedures were performed in one surgery

Intraoperative fluid management plan ('after' cohort)

Goal for fluid management:

Mean Arterial Pressure >= 65 mm hg, pulse rate < 90 bpm, urinary output < 0.5 ml/kg/h, hemoglobin concentration >= 9 g/dl, Sentral venous oxygen saturation >= 70, pulse pressure variation < 10.

1. Fluid bolus 500 ml Ringers acetat. Evaluate (see Goal) after 15 minutes
2. Repeat fluid bolus until Goal is reached
3. Fluid aspirated from nasogastric tube should be replaced (1:1) with intravenous supplement of saline (NaCl 154 mmol/L)
4. If Goal is not reached after 2000 – 4000 ml of crystalloid fluid consider infusion of kolloid (Albumine)

Table S3: Intraoperative fluid therapy

Intervention	'After' cohort	'Before' cohort	*P*-value
**Intraoperative fluid therapy, volume (ml), mean, SD**			
Krystalloid	2347 (1043)	2300 (1099)	0.70[c]
Kolloids	83 (242)	63 (157)	0.27[b]
Erythrocyte infusion	57 (288)	45 (184)	0.83[b]
Trombocyte infusion	7(51)	1(15)	0.27[b]

[a] Mann–Whitney test

[b] Chi-squared test

[c] *t*-test

Table S4: Adherence to bundle care elements

Intervention	‘After’ cohort	‘Before’ cohort	*P*-value
**Preoperative variables, n (%)**			
Specialist involved in case, n (%)			< 0.001[b]
Surgeon only	13 (8)	54 (32)	
Anaesthetist only	14 (9)	21 (13)	
Surgeon and anaesthetist	123 (80)	77 (46)	
Junior doctor only	4 (3)	16 (10)	
Missing data	0	2	
**Intraoperative variables, n (%)**			
Anesthesia depth monitoring	131 (85)	5 (3)	< 0.001[b]
Perop norepinephrine, n (%)	153 (99)	155 (91)	
Perop phenylephrine use, n (%)	1 (1)	15 (9)	< 0.001[b]
**Postoperative variables**			
LOS in PACU (hours), median (Q1-Q3)	21 (15-28)	17 (9-24)	0.004[a]
Postoperative physiotherapy, n (%)	127 (83)	132 (78)	0.28[b]
Hb ≥ 9 g/dl (intra- and postoperatively), n (%)	97 (63)	117(69)	0.31[b]

[a] Mann–Whitney test

[b] Chi-squared test

[c] *t*-test

Sample size considerations

The following table shows the distribution of CCI scores in the historical data and the postulated distribution corresponding to an approximate 25% reduction in mean scores. The means for the historical and postulated data were 41.7 and 30.9, respectively. (The exact calculations were done using more decimals than shown in the table.)

Based on this, we ran computer simulations to calculate the power for both the Mann–Whitney test, Welch's *t*-test and a chi-squared test, the latter just comparing mortality, i.e., a CCI score of 100. All simulations were run using a significance level of 5%.

Our target was a power of at least 90%. The simulations showed that using mortality as the primary outcome would lead to a very large sample size (1,051 patients in each group), which was infeasible for a single-centre study. They also showed that the Wilcoxon–Mann–Whitney test in general had the greatest statistical power, and by including 140 patients in each group we would get a power of 90% using this test. (The corresponding power for the *t*-test and the chi-squared test was 74% and 22%, respectively).

We therefore decided to aim for at least 140 patients in the 'after' cohort and to analyse the primary outcome using the Mann–Whitney test.

	**Historic**		**Postulated**
**CCI**	***n***	**Prop.**	**Prop.**
0	15	14.15%	20.02%
8.7	10	9.43%	13.01%
12.2	1	0.94%	7.01%
20.9	9	8.49%	11.01%
22.6	4	3.77%	7.01%
24.2	2	1.89%	2.00%
29.6	4	3.77%	4.00%
30.8	10	9.43%	9.01%
32.0	2	1.89%	2.00%
33.7	1	0.94%	0.00%
35.9	1	0.94%	0.00%
36.2	9	8.49%	5.01%
37.2	4	3.77%	2.00%
39.7	1	0.94%	0.00%
42.4	1	0.94%	0.00%
42.7	1	0.94%	0.00%
43.6	1	0.94%	0.00%
45.7	2	1.89%	0.90%
66.0	1	0.94%	0.00%
73.6	1	0.94%	0.00%
77.6	1	0.94%	0.00%
82.8	1	0.94%	0.00%
100	24	22.64%	17.02%
**Total**	**106**	**100%**	**100%**
